# Evaluation of RNA Blood Biomarkers in the Parkinson’s Disease Biomarkers Program

**DOI:** 10.3389/fnagi.2018.00157

**Published:** 2018-05-29

**Authors:** Jose A. Santiago, Virginie Bottero, Judith A. Potashkin

**Affiliations:** Department of Cellular and Molecular Pharmacology, The Chicago Medical School, Rosalind Franklin University of Medicine and Science, North Chicago, IL, United States

**Keywords:** Parkinson’s disease, progressive supranuclear palsy, biomarker, blood, PDBP

## Abstract

There is a high misdiagnosis rate between Parkinson’s disease (PD) and atypical parkinsonian disorders (APD), such as progressive supranuclear palsy (PSP), the second most common parkinsonian syndrome. In our earlier studies, we identified and replicated RNA blood biomarkers in several independent cohorts, however, replication in a cohort that includes PSP patients has not yet been performed. To this end, we evaluated the diagnostic potential of nine previously identified RNA biomarkers using quantitative PCR assays in 138 blood samples at baseline from PD, PSP and healthy controls (HCs) nested in the PD Biomarkers Program. Linear discriminant analysis showed that *COPZ1* and *PTPN1* distinguished PD from PSP patients with 62.5% accuracy. Five biomarkers, *PTPN1*, *COPZ1*, *FAXDC2*, *SLC14A1s* and *NAMPT* were useful for distinguishing PSP from controls with 69% accuracy. Several biomarkers correlated with clinical features in PD patients. *SLC14A1-s* correlated with Unified Parkinson’s Disease Rating Scale total and part III scores. In addition, *COPZ1*, *PTPN1* and *MLST8*, correlated with Montreal Cognitive Assessment (MoCA). Interestingly, *COPZ1*, *EFTUD2* and *PTPN1* were downregulated in cognitively impaired (CI) compared to normal subjects. Linear discriminant analysis showed that age, *PTPN1*, *COPZ1*, *FAXDC2*, *EFTUD2* and *MLST8* distinguished CI from normal subjects with 65.9% accuracy. These results suggest that *COPZ1* and *PTPN1* are useful for distinguishing PD from PSP patients. In addition, the combination of *PTPN1*, *COPZ1*, *FAXDC2*, *EFTUD2* and *MLST8* is a useful signature for cognitive impairment. Evaluation of these biomarkers in a larger study will be a key to advancing these biomarkers into the clinic.

## Introduction

Parkinson’s disease (PD) is a neurodegenerative disease characterized by the selective loss of dopamine neurons in the substantia nigra pars compacta. Deterioration of the dopaminergic system leads to severe motor symptoms including resting tremor, rigidity, bradykinesia and postural instability. Current treatments for PD afford symptomatic relief, but a disease modifying or neuroprotective agent capable of halting the progression of the disease is not yet available. The lack of a robust biomarker with high sensitivity and specificity has limited the progress towards the development of effective therapeutics for PD. In this context, biomarkers would offer great advantages in clinical trials testing drugs and neuroprotective agents. For example, biomarkers could facilitate the selection and stratification of study participants, monitor disease progression and inform about target selection (Santiago and Potashkin, 2014a; Gwinn et al., 2017).

Distinguishing PD from atypical parkinsonian disorders (APD) is an unmet goal for currently proposed biomarker studies. The overlap in symptoms and pathological features between PD and APD makes these diseases very difficult to distinguish early in the disease process where therapeutic intervention may be more beneficial (Rajput and Rajput, 2014; Santiago and Potashkin, 2014c). Progressive supranuclear palsy (PSP), for example, is frequently misdiagnosed as PD. Deposition of fibrillar aggregates of four-repeat Tau protein in the brainstem and cerebral cortex is a pathological hallmark of PSP (Dickson et al., 2010). Clinical symptoms in PSP patients include prominent hypokinesia, oculo-motor and balance disturbances (Dickson et al., 2010). In contrast, PD is characterized by the accumulation of alpha synuclein (SNCA) in the substantia nigra pars compacta. PD patients exhibit classical motor symptoms that include rigidity, tremor and bradykinesia (Ascherio and Schwarzschild, 2016). To date, diagnosis of PD and PSP patients is based on the assessment of motor symptoms and response to dopaminergic therapy (Ascherio and Schwarzschild, 2016). The problem with this approach is that PSP and PD patients manifest similarities at early stages of the disease and both respond to dopaminergic treatment, which makes the diagnosis very challenging (Dickson et al., 2010). The high misdiagnosis rate between PD and PSP reaching approximately 30% heightens the urgency for the identification of highly specific biomarkers capable of distinguishing these diseases (Rajput and Rajput, 2014; Santiago and Potashkin, 2014c).

Although substantial progress has been made in the discovery of blood biomarkers for PD (Khoo et al., 2012; Ciaramella et al., 2013; Qiang et al., 2013; Santiago and Potashkin, 2013a, 2014a, 2015b, 2017; Santiago et al., 2014, 2016; Alieva et al., 2015; Calligaris et al., 2015; Locascio et al., 2015; Swanson et al., 2015), very few studies have addressed the misdiagnosis problem between PD and PSP. Our first studies identified a splice-variant specific signature capable of distinguishing PD from healthy and APD (Potashkin et al., 2012). This biomarker signature was composed of 13 splice variants including fatty acid hydroxylase domain containing 2 (*FAXDC2; C5ORF4)*, coatomer protein complex subunit zeta 1 (*COPZ1*), microtubule-actin crosslinking factor 1 (*MACF1*), wntless WNT ligand secretion mediator (*WLS*), proteoglycan 3, pro eosinophil major basic protein 2 (*PRG3*), zinc finger protein 160 (*ZNF160*), elongation factor Tu GTP binding domain containing 2 (*EFTUD2*), mitogen-activated protein 4 kinase 1 (*MAP4K1*), membrane palmitoylated protein 1 (*MPP1*), pyruvate kinase M2 (*PKM2*), solute carrier family 14 member 1 (*SLC14A1-s*), *SLC14A1-l* and zinc finger protein 134 (*ZNF134*; Potashkin et al., 2012). Seven out these 13 biomarkers, including* FAXDC2 (C5ORF4)*, *COPZ1*, *MACF1*, *WLS, PRG3*, *ZNF160* and *EFTUD2*, replicated in a second independent cohort of participants that included PD and healthy controls (HCs), but not APD patients (Santiago et al., 2013). Another promising study used a network approach integrating our microarray data (Potashkin et al., 2012) in order to identify protein tyrosine phosphatase, non-receptor type 1 (*PTPN1*) as a potential biomarker for PSP (Santiago and Potashkin, 2014c). *PTPN1* was capable of distinguishing PD from PSP patients with 86% overall diagnostic accuracy. Nonetheless, these markers have not been replicated in an independent set of participants that includes PSP patients. In this study, we tested a subset of nine RNA biomarkers in blood samples obtained from the PD Biomarkers Program (PDBP). We hypothesized that these RNA markers from our previous studies could be helpful for the differential diagnosis between PD and PSP and may be informative of clinical features including disease progression and cognition.

## Materials and Methods

### Study Participants

Samples used in this study were obtained from PDBP, a consortium of clinical sites funded by the National Institute of Neurological Diseases and Stroke (NINDS, National Institutes of Health (NIH), United States). The consortium projects focus on the development of clinical and laboratory-based biomarkers for PD diagnosis, progression and prognosis. RNA samples used in this study were obtained from Penn State Milton S. Hershey Medical Center and University of Florida College of Medicine. The Institutional Review Boards (IRB) of each PDBP center and the Rosalind Franklin University of Medicine and Sciences approved the study protocol. Written informed consent was obtained from all participants before inclusion in the study. PD patients were recruited and evaluated by movement disorder specialists using establish criteria (Rosenthal et al., 2016). Disease severity was assessed using the Movement Disorder Society Unified Parkinson’s Disease Rating Scale (MDS-UPDRS) part III and Hoehn & Yahr scale. Inclusion and exclusion criteria were the following: PD patients had a history of adequate response to dopaminergic therapy and history of asymmetrical symptom onset. HC had no history of neurological disorder; PSP patients were over 40 years old with vertical gaze palsy and/or slow vertical gaze/postural instability during first year of diagnosis. Additional inclusion and exclusion criteria have been published elsewhere (Rosenthal et al., 2016). The demographic and clinical characteristics of the study participants selected for this study are listed in Table 1.

**Table 1:** Demographic and clinical characteristics of Parkinson’s disease Biomarkers Program (PDBP) participants.

Characteristic	HC (*n* = 50)	PD (*n* = 48)	PSP (*n* = 40)	*P* value^a^ (HC/PD)	*P* value^a^ (HC/PSP)	*P* value^a^ (PD/PSP)
Age, mean (SD) [95% CI], years	69 (6) [68–71]	70 (6) [68–72]	70 (7) [68–72]	0.91	0.97	0.89
Age of onset, mean (SD) [95% CI], years	N.A.	64 (8) [60–66]	68 (8) [65–70]	N.A.	N.A.	0.02
Female/male, No. (%male)	25/25 (50)	25/23 (48)	20/20 (50)	0.90^b^	0.90^b^	0.90^b^
Disease duration, median (range), years	N.A.	6.3 (0.17–25)	2.5 (0.17–9.2)	N.A	N.A	<0.0001
Hoehn & Yahr stage, mean (SD)	0.10 (0.36)	2.04 (0.96)	2.35 (1.81)	<0.0001	<0.0001	0.34
MDS-UPDRS total, mean (SD) [95% CI]	6.48 (6.6) [4.5–8.3]	50.33 (41.3) [3.3–62.3]	57.23 (33.5) [46.5–67.9]	<0.0001	<0.0001	0.39
MDS-UPDRS part III	4.58 (5.8) [2.92–6.24]	33.60 (22.2) [27.1–40.1]	46.93 (18.4) [41.0–52.8]	<0.0001	<0.0001	0.0028
MoCA, mean (SD) [95% CI]	26.20 (2.1) [25.6–26.8]	23.11 (3.6) [22–24.2]	20.32 (4.9) [18.6–21.9]	<0.0001	<0.0001	0.006

*Abbreviations: CI, 95% confidence interval; HC, healthy controls; MoCA, Montreal Cognitive Assessment; MDS-UPDRS, Unified Parkinson’s Disease Rating Scale; PD, Parkinson’s disease; PSP, progressive supranuclear palsy; SD, standard deviation; y, years. ^a^Based on a Student t-test. ^b^Based on chi-square test (X^2^)*.

### RNA Samples

We obtained a total of 138 RNA samples at baseline from age and sex-matched early stage PD, PSP patients and HC from PDBP. Standardized clinical and biospecimen collection procedures were used for all participants. Each biospecimen collection follows the PDBP protocol and mirrors the Alzheimer’s Disease Neuroimaging Initiative (ADNI), BioFIND and PPMI protocols. Collected biosamples are sent to the NINDS Repository (Coriell Laboratories), undergo quality control, and are cataloged. Blinded RNA samples were shipped in dry ice to Rosalind Franklin University of Medicine and Sciences for the studies described herein.

### Quantitative Polymerase Chain Reaction Assays

Samples with RNA integrity values >6.0 and absorbance 260/280 between 1.8 and 2.4 were used in this study. 0.4 microgram of RNA was reverse transcribed into cDNA using a mix of random hexamer primers (High Capacity cDNA Synthesis Kit, Life Technologies, Carlsbad, CA, USA). Quantitative polymerase chain reaction assays (qRT-PCR) were performed using ViiA7 real-time PCR system (Thermo Fisher Scientific, Waltham, MA, USA). Each 25 microliters reaction contained Bullseye EvaGreen qPCR 2X Mastermix (MIDSCI, St. Louis, USA) and primers at a concentration of 0.05 mM. Primer sequences are indicated in Table 2. Amplification steps used were as follows: denature at 95°C for 10 min, annealing at 95°C for 15 s extension at 60°C for 45 cycles of amplification and 95°C extension for 15 s Melting curve was performed using the following conditions: 60°C for 1 min following by 5°C/s and 95°C for 15 s qRT-PCR assays were performed in triplicates. The geometric mean of the two reference genes, glyceraldehyde-3-phosphate dehydrogenase (*GAPDH*) and actin beta (*ACTB*), were used to normalize for input RNA. Expression data was analyzed using the comparative ΔΔCt method. In this method, the amount of target is normalized to an endogenous reference and relative to a calibrator.

**Table 2:** Primer sequences for the biomarkers tested in PDBP.

	Biomarker	Forward	Reverse
1	*GAPDH*	CAACGGATTTGGTCGTATTGG	TGATGGCAACAATATCCACTTTACC
2	*ACTB*	TCACCCACACTGTGCCATCTACGA	CAGCGGAACCGCTCATTGCCAATGG
3	*COPZ1*	GATTTTGTGGTGGGAAAGAGT	TGACAGCTCCCCTAGATCTTTG
4	*FAXDC2 (C5ORF4)*	GACATGGTGGATCCTGTGAAACT	GAAAGATATCATGCACTGGTTGAAA
5	*SLC14A1s*	CACTCATGTGCCTGCATGCT	AACAGGGCCGCTGCTATG
6	*COPS7A*	ATGAGTGCGGAAGTGAAGGTG	GCTCTCTAACATTGGGCATGTC
7	*MLST8*	ATCCGCATGTATGATCTCAACTC	CCACAGACGCGATGTTCTTG
8	*PTPN1*	AAGAACAAAAACCGAAATAGGTACAGA	CCAAAAGTGACCGCATGTGT
9	*NAMPT*	CTATAAACAATATCCACCCAACACAAG	GTTTCCTCATATTTCACCTTCCTTAATT
10	*EFTUD2*	AGCAGGCGAGAGATGGATGA	CGGCTGTTGGGTAGTACTTCTTG
11	*PTBP1*	GCTCAGGATCATCGTGGAGAA	ATCTTCAACACTGTGCCGAACTT

### Statistical Analysis

Statistical analyses were performed using STATISTICA 12 (StatSoft, OK, USA) and GraphPad Prism version 5 (GraphPad Software Inc., CA, USA). A Student-*t*-test (unpaired, two tailed) was used to assess the differences between two groups and a chi-square test was used to analyze categorical data. Correlation analysis was performed using the Pearson method. A Bonferroni corrected *p*-value of 0.005 or less was regarded as significant. A forward stepwise linear discriminant analysis was performed to determine the variables that best discriminated between groups as described previously (Potashkin et al., 2012). Biomarker performance was assessed using a receiver operating characteristic curve (ROC) analysis. All the data used in preparation of this manuscript is publicly available at: https://pdbp.ninds.nih.gov/.

## Results

### Demographic and Clinical Characteristics of Study Participants

There were no significant differences in mean age and sex distribution between PD, PSP and HC (Table 1). PD patients had a significantly lower age of onset (64 vs. 68 years old) and a higher disease duration (6.3 vs. 2.5 years) compared to PSP patients. There were no significant differences in Hoehn & Yahr (2.04 vs. 2.35) and MDS-UPDRS total scores (50.33 vs. 57.23) between PD and PSP patients. Analyses of the clinical metrics to assess PD staging and cognition, MDS-UPDRS part III and Montreal Cognitive Assessment (MoCA), showed some differences between PD and PSP patients. For example, PSP patients exhibited significantly higher MDS-UPDRS part III (46.93 vs. 33.60) and lower MoCA scores (20.32 vs. 23.11) compared to PD patients (Table 1).

### Evaluation of RNA Biomarkers in PDBP Study Participants

A subset of nine RNA biomarkers were tested in 50 HC, 48 PD and 40 PSP blood samples at baseline from individuals nested in the PDBP cohort by qRT-PCR assays (Table 2). Seven out of the nine biomarkers including *COPZ1*, *FAXDC2*, *EFTUD2, SLC14A1s*, *PTPN1*, nicotinamide phosphoribosyl transferase (*NAMPT*) and polypyrimidine tract binding protein 1 (*PTBP1*) were tested in our previous studies (Potashkin et al., 2012; Santiago et al., 2013, 2016; Santiago and Potashkin, 2014c, 2015a,b). In this study, we also tested two additional biomarkers, COP9 signalosome subunit 7A (*COPS7A*) and MTOR associated protein, LST8 homolog (*MLST8*). These markers were selected from a ranked list of potential candidates identified in a network analysis for PSP (Santiago and Potashkin, 2014c). Several biomarkers showed significant correlations with clinical features. For example, *SLC14A1-s* mRNA correlated with MDS-UPDRS total (*r* = −0.18, *p* = 0.03) and MDS UPDRS part III scores (*r* = −0.18, *p* = 0.03). Expression levels of three biomarkers, *COPZ1* (*r* = 0.21, *p* = 0.01), *PTPN1* (*r* = 0.18, *p* = 0.03) and *MLST8* (*r* = 0.18, *p* = 0.01) correlated with MoCA scores.

We next investigated the capacity of each biomarker for distinguishing between the following groups: PD vs. PSP, PD vs. HC, and PSP vs. HC. Analysis of each biomarker alone did not reach significance after adjusting for multiple comparisons (*p* < 0.005; Supplementary Table S1). Of note, *COPZ1* (*p* = 0.008) and *PTPN1* (*p* = 0.008) trended toward significance when comparing PD vs. HC but failed to reach significance after adjusting for multiple comparisons. We performed a linear discriminant analysis to assess whether combination of biomarkers could be useful for discriminating between PD vs. HC and PSP vs. HC. Linear discriminant analysis showed that *PTPN1* and *COPZ1* were the only markers retained in the model capable of discriminating PD from HC with 58% overall diagnostic accuracy (Table 3). Evaluation of biomarker performance by receiver operating characteristic curve analysis (ROC) resulted in an area under the curve (AUC) value of 0.56. The same analysis was performed to identify the best group of biomarkers for discriminating PSP from HC. In this analysis, five biomarkers, *PTPN1*, *COPZ1*, *FAXDC2*, *SLC14A1s* and *NAMPT* were identified as the best group of markers for distinguishing PSP from HC with an overall diagnostic accuracy of 69% (Table 3). ROC analysis resulted in an AUC value of 0.66.

**Table 3:** Linear discriminant analysis for RNA PD biomarkers tested in PDBP.

	PD vs. PSP	PD vs. HC	PSP vs. HC	CI vs. CN
Variables retained in the model	*COPZ1* *PTPN1*	*COPZ1* *PTPN1*	*COPZ1* *PTPN1* *FAXDC2* *SLC14A1s* *NAMPT*	*COPZ1* *PTPN1* *FAXDC2* *EFTUD2* *MLST8* Age
Variables removed from the model	*EFTUD2* *FAXDC2* *SLC14A1s* *NAMPT* *PTBP1* *MLST8* *COPS7A* Age Sex	*EFTUD2* *FAXDC2* *SLC14A1s* *NAMPT* *PTBP1* *MLST8* *COPS7A* Age Sex	*EFTUD2* *PTBP1* *MLST8* *COPS7A* Age Sex	*SLC14A1s* *NAMPT* *PTBP1* *COPS7A* Sex
Overall diagnostic accuracy (%)	62.5%	58%	69%	65.9%

*A forward step-wise linear discriminant analysis was performed to identify the best group of variables for discriminating between groups. CI, cognitively impaired; CN, cognitively normal; HC, healthy controls; PSP, progressive supranuclear palsy; PD, Parkinson’s disease*.

Similarly, we analyzed which biomarkers in combination could be useful for distinguishing PD from PSP. Linear discriminant analysis showed that two biomarkers, *COPZ1* and *PTPN1*, were retained in the model as useful for discriminating PD from PSP patients. Specifically, *PTPN1* and *COPZ1* together achieved an overall diagnostic accuracy of 62.5% (Table 3). The remaining variables including age, sex, *FAXDC2*, *NAMPT*, *SLC14A1s*, *PTBP1, EFTUD2*, *COPS7A* and *MLST8* did not contribute to the discrimination between the groups and were therefore eliminated from the model. ROC analysis resulted in an AUC value of 0.60.

### RNA Markers Associated With Cognitive Decline

**Figure 1:**
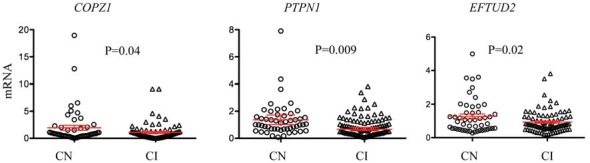
RNA biomarkers associated with cognitive decline. Relative abundance of *COPZ1*, *PTPN1* and *EFTUD2* was compared in subjects with normal cognition (CN = 51, circles) to cognitively impaired (CI = 87, triangles). Error bars represent the 95% confidence interval (CI). A *p*-value of less than 0.05 was regarded as significant based on a Student *t*-test (two-tailed).

Since some of the biomarkers correlated with MoCA, a standard clinical measure for cognitive performance, we investigated the potential of each biomarker for identifying cognitively impaired (CI) subjects. Cognitive performance was defined using the MoCA cutoff lower than 26 for cognitive impairment as previously described (Santiago and Potashkin, 2015a; Weintraub et al., 2015). There were 51 cognitively normal (CN) and 87 CI participants in this subset of PDBP. Three biomarkers, *COPZ1*, *PTPN1* and *EFTUD2* were significantly downregulated in CI compared to CN individuals (Figure 1). Evaluation of biomarker performance by ROC analysis resulted in AUC values of 0.57 for *COPZ1*, 0.57 for *EFTUD2* and 0.64 for *PTPN1*. Combination of these three markers resulted in AUC value of 0.65. We next performed a linear discriminant analysis to determine which variables best distinguished between CI and CN. This analysis showed that six variables including age, *PTPN1*, *COPZ1*, *FAXDC2*, *EFTUD2* and *MLST8* were retained in the model as the best discriminant factors (Table 3). Using these six variables CI subjects were distinguished from CN with an overall 65.9% accuracy. Sex, *COPS7A*, *NAMPT*, *SLC14A1s*, and *PTBP1* did not contribute to the discrimination between the groups and were therefore eliminated from the model. ROC analysis resulted in AUC value of 0.63.

## Discussion

Replication and validation of biomarker studies in PD is essential for the translation of biomarkers into useful diagnostic tools for researchers and clinicians. Several biomarkers have shown promise for identifying early stage PD patients, however, very few studies have been replicated in independent clinical cohorts or addressed the misdiagnosis problem in PD. Specifically, only a handful of studies have evaluated the potential of biomarkers in distinguishing PD from APD (Potashkin et al., 2012; Santiago and Potashkin, 2014c; Hansson et al., 2017). In this study, we evaluated the diagnostic and prognostic potential of previously identified RNA blood biomarkers for PD in samples nested in PDBP. Replication in a well-characterized cohort like PDBP that included PSP patients was important to assess the performance of our biomarkers in distinguishing PD from APD, which remains an unmet need in the field.

The primary objective of this study was to replicate previously identified RNA biomarkers in an independent set of samples that includes PSP patients. We tested a subset of biomarkers including *FAXDC2 (C5ORF4)*, *COPZ1*, *EFTUD2*, *SLC14A1-s*, *HNF4A*, *PTBP1* and *PTPN1*. The biomarkers evaluated in this study have been implicated in pathways involved in the pathogenesis of PD. For example, *FAXDC2* is a member of the fatty acid hydrolase superfamily known to be involved in cholesterol metabolism, which has been shown to contribute to neurodegeneration in PD (Paul et al., 2017). *COPZ1* encodes a subunit of the cytoplasmic coatomer complex involved in autophagy and protein trafficking (Santiago and Potashkin, 2015a; Bensellam et al., 2016). *EFTUD2* encodes the splicing factor U5–116 kD and it may play a role in RNA splicing during neural development (Lei et al., 2017). In this context, aberrant alternative splicing in blood of PD patients have been reported in numerous studies (Potashkin et al., 2012; Santiago et al., 2013; Alieva et al., 2014). In addition, *COPZ1* and *EFTUD2* have been associated with cognitive decline in PD patients (Santiago and Potashkin, 2015a). Other biomarkers including hepatocyte nuclear factor 4 alpha (*HNF4A*), *PTBP1* and *PTPN1* have been implicated in glucose metabolism and insulin regulation, biological processes that have been extensively associated with the pathogenesis of PD (Knoch et al., 2006; Santiago and Potashkin, 2013b, 2015a). We also tested two additional biomarkers, *MLST8* and *COPS7A* that were identified in our previous study on PSP (Santiago and Potashkin, 2014c). MLST8 is a subunit of the mammalian target rapamycin complexes 1 and 2 (mTORC1, mTORC2) known to regulate mTOR kinase activity (Kim et al., 2003) which has been involved in a neuroprotective mechanism in PD (Malagelada et al., 2010). *COPS7A* encodes a component of the COP9 signalosome, a protein complex involved in the ubiquitin conjugation pathway, protein misfolding and autophagy (Liu et al., 2016), pathways implicated in the development of PD (Cook et al., 2012).

First, we analyzed the capacity of each biomarker independently to distinguish between groups (PD vs. HC, PD vs. PSP and PD vs. PSP). None of the biomarkers alone reached statistical significance after adjusting for multiple comparisons. Notably, only two biomarkers, *COPZ1* and *PTPN1* trended toward significance (*p* = 0.008) but failed to reach significance after adjusting for multiple comparisons. Despite these results, it is important to note that these markers have been replicated in samples from several independent cohorts. For instance, *COPZ1* has been replicated in two cohorts of medicated PD patients (Potashkin et al., 2012; Santiago et al., 2013) and in a cohort of drug-naïve PD patients (Santiago and Potashkin, 2015a).

We next investigated whether combination of biomarkers could be useful for distinguishing between groups. To this end, we performed a linear discriminant analysis as previously described (Potashkin et al., 2012; Santiago et al., 2013). Based on the discriminant analysis, two biomarkers, *COPZ1* and *PTPN1* were capable of distinguishing PD from PSP patients while the remaining biomarkers were excluded from the model. The overall accuracy obtained with these two biomarkers was 62.5%. This accuracy is less than that obtained in our previous studies. For example, our original splice variant signature distinguished PD from APD patients with 95% diagnostic accuracy in samples obtained from the Diagnostic and Prognostic Biomarkers for Parkinson’s Disease (PROBE) study (Potashkin et al., 2012). However, the APD group in PROBE was comprised of multiple system atrophy and PSP patients, whereas the present study only includes PSP patients. Further, *PTPN1* alone achieved an overall diagnostic accuracy of 86% in discriminating PD from PSP patients in samples obtained from PROBE (Santiago and Potashkin, 2014c). The discrepancy in the results may be explained by several differences in the clinical studies including, patient population and protocols for sample collection and storage.

Analysis of biomarker expression in PD patients compared to HC showed that *COPZ1* and *PTPN1* were the only two biomarkers selected with the highest discriminant power for distinguishing between groups with a 58% overall diagnostic accuracy. Similarly, we also determined which biomarkers combined could distinguish PSP from HC. Five biomarkers including *PTPN1*, *COPZ1*, *FAXDC2*, *SLC14A1s* and *NAMPT* distinguished PSP from HC with a 69% diagnostic accuracy. Although this result is encouraging, the real challenge is not to distinguish PSP from HC but rather finding specific and sensitive biomarkers that facilitate the differential diagnosis between PD and PSP. Collectively, these results suggest that these markers alone or in combination do not possess the ideal diagnostic capacity required in a robust biomarker for distinguishing PD from PSP. Combination of multiple biomarkers including, protein, RNA, imaging and other clinical tests will be required to build a diagnostic model for PD. In this regard, blood neurofilament light chain (NfL) protein distinguished PD from APD patients including PSP, MSA and corticobasal syndrome with high accuracy, 91% specificity and 82% sensitivity (Hansson et al., 2017). In addition, imaging techniques have achieved high sensitivity and specificity in distinguishing PD from APD. For example, free-water derived from diffusion magnetic resonance imaging (MRI) has been useful for detecting differences in the substantia nigra of PD patients compared to PSP, MSA and HC (Planetta et al., 2016; Ofori et al., 2017). Thus, combining *COPZ1* and *PTPN1* mRNAs with protein markers like NfL and/or imaging markers could improve the diagnostic accuracy of PD and PSP patients. In addition to these markers, there are other interesting molecular signatures that have shown promise in distinguishing PD from HC and other neurodegenerative diseases that could be tested in samples from PSP patients. For example, a molecular signature in blood composed of five genes, p19 S-phase kinase-associated protein 1A (*SKP1A*), huntingtin interacting protein-2 (*HIP2*), aldehyde dehydrogenase family 1 subfamily A1 (*ALDH1A1*), 19 S proteasomal protein (*PSMC4*) and heat shock 70-kDa protein 8 (*HSPA8*), distinguished early-stage and *de novo* PD from HC and Alzheimer’s disease patients with 90.3% sensitivity and 89.1% specificity (Molochnikov et al., 2012). Transcriptomic profiling of blood employing RNA sequencing is a robust method for finding additional candidate biomarkers. In this context, RNA sequencing analysis revealed gene expression changes in blood of PD patients before and after deep brain stimulation treatment (Soreq et al., 2013, 2015a,b). These high-throughput technologies combined with bioinformatic analyses including weighted gene coexpression networks have been useful for identifying changes in gene expression of both protein-coding and small and long regulatory RNAs (lncRNAs) in neurodegenerative diseases (Guffanti et al., 2014; Santiago and Potashkin, 2014b). For instance, RNA sequencing analysis revealed over 3000 lncRNAs coexpressed in both brain and blood of PD patients (Soreq et al., 2014). Therefore, a comprehensive analysis of the blood transcriptome using RNA sequencing and network analyses will be key for identifying biologically relevant biomarkers for PSP. Further, weighted gene co-expression networks analysis in both brain and blood datasets will be useful for elucidating potential mechanisms of disease pathogenesis in PSP (Guffanti et al., 2014; Soreq et al., 2014).

Cognitive impairment is a disabling non-motor symptom frequently observed in PD patients. The decline in cognitive performance has been documented in *de novo* and untreated PD patients suggesting cognitive impairment may be one of the earliest manifestations in the development of PD (Santiago and Potashkin, 2015a; Weintraub et al., 2015). Identifying predictors and biomarkers of cognitive impairment is expected to be valuable in patient classification, stratification and personalized treatment (Mollenhauer et al., 2014). In this study, three biomarkers, *COPZ1*, *PTPN1* and *MLST8* correlated with MoCA, although the correlation values were low. Further analysis showed that three biomarkers *COPZ1*, *EFTUD2* and *PTPN1* were significantly downregulated in CI subjects compared to normal cognition suggesting these biomarkers may be useful to assess cognitive performance in patients. Evaluation of biomarker performance by ROC analysis resulted in AUC values of 0.57 for *COPZ1*, 0.57 for *EFTUD2* and 0.64 for *PTPN1* indicating a modest diagnostic accuracy. Combination of these three markers together resulted in AUC value of 0.65. The AUC value for *PTPN1* alone resulted in 0.64, therefore combination of the three markers did not improve substantially the diagnostic accuracy. We next sought to build a diagnostic model for cognitive impairment. Discriminant analysis showed that the best predictors of cognitive decline were *EFTUD2*, *COPZ1*, *PTPN1*, *FAXDC2*, *MLST8* and age. This signature distinguished cognitive impairment from CN subjects with 66% accuracy. In this regard, two of these biomarkers, *EFTUD2* and *PTBP1* correlated with MoCA scores in drug naïve PD patients obtained from the Parkinson’s Progression Markers Initiative (PPMI; Santiago and Potashkin, 2015a). Further analysis showed that relative expression of both *EFTUD2* and *PTBP1* was significantly downregulated in PD patients with cognitive impairment compared to PD patients with normal cognition (Santiago and Potashkin, 2015a). Thus, these results confirm the association of *EFTUD2* with cognitive impairment in a second independent cohort. Evaluation of the other markers included in the signature in a larger and well-characterized cohort of participants is warranted. In addition, combination of these RNA markers with protein biomarkers previously identified as predictors of cognitive performance like epidermal growth factor (EGF) and insulin like growth factor 1 (IGF-1) may improve the diagnostic accuracy and patient stratification (Chen-Plotkin et al., 2011; Pellecchia et al., 2014). In addition, given the presence of cognitive impairment in dementia with Lewy bodies, vascular dementia, vascular parkinsonism and PD dementia (Wen et al., 2017; Jellinger and Korczyn, 2018), it will be important to assess the utility of these markers in distinguishing between the different dementia subtypes.

There are several important aspects in this study design that need to be considered when interpreting the results from this study and its discrepancies with our previous studies. For example, PDBP differs from PPMI in that PD patients enrolled in the latter were *de novo* patients not taking PD medications at the time of enrollment. Dopaminergic treatment can certainly affect gene expression and introduce bias since control groups are exposed to different medications. To ensure consistency and allow the successful replication and validation of biomarker studies, standardized protocols for RNA extraction, sample processing and storage must be followed among all clinical sites.

Given the complex heterogeneity in PD clinical subtypes it has become evident that a single biomarker may not be useful as a diagnostic tool for PD and APD. Multimodal biomarker approaches including molecular markers, imaging and clinical tests may be the route to improve the diagnosis and the clinical management of PD patients. Further, the different PD clinical subtypes and comorbidities associated with PD should be considered in the design of future biomarker studies (Santiago et al., 2017). Replication and validation of biomarkers studies in independent cohorts as well as the dissemination of positive and negative results should be encouraged among researchers in order to advance the development of diagnostic strategies for PD.

## Author Contributions

JS and JP: conceived and designed the experiments. JS and VB: performed experiments and wrote the article. JS, VB and JP: analyzed the data.

## Conflict of Interest Statement

The authors declare that the research was conducted in the absence of any commercial or financial relationships that could be construed as a potential conflict of interest.
