# Supplementary material for: Evaluation of RNA Blood Biomarkers in the Parkinson’s Disease Biomarkers Program
Source: Front Aging Neurosci. 2018 May 29;10:157. doi: 10.3389/fnagi.2018.00157 (PMC5986959; doi:10.3389/fnagi.2018.00157)
Supplement: Supplementary file 1 [file Table_1.DOC]

**Supplementary table 1.** Relative expression levels of biomarkers in PDBP. P-values are assessed by Student t-test. CI is 95% confidence interval, S.E is standard error. A p-value of ≤ 0.005 was regarded significant.

| **Biomarker** | **Difference between means (PD vs PSP)**  **(S.E) [95% CI], p-value** | **Difference between means**  **(PD vs HC)**  **(S.E) [95% CI], p-value** | **Difference between means**  **(PSP vs HC)**  **(S.E) [95% CI], p-value** |
| --- | --- | --- | --- |
| *COPZ1* | -0.28 (0.25)  [-0.79-0.23], p=0.13 | 1.01 (0.38)  [0.26-1.75], p=0.008 | 0.72 (0.46)  [-0.19-1.64], p=0.11 |
| *PTPN1* | 0.19 (0.16)  [-0.13-0.52],p =0.12 | 1.04 (0.12)  [-0.14-0.72], p=0.008 | 0.48 (0.21)  [0.05-0.02], p=0.02 |
| *FAXDC2* | -0.02 (0.41)  [-0.85-0.80], p=0.47 | -0.18 (0.39)  [-0.96-0.59], p=0.32 | -0.21 (0.39)  [-0.99-0.56], p=0.58 |
| *SLC14A1s* | 0.13 (0.17)  [-0.21-0.47], p=0.22 | 0.36 (0.25)  [-0.15-0.87], p=0.09 | 0.49 (0.25)  [-0.02- 0.99, p=0.05 |
| *EFTUD2* | 0.04 (0.14)  [-0.24-0.32], p=0.76 | 0.23 (0.18)  [-0.12-0.60], p=0.19 | 0.28 (0.18)  [-0.08-0.64], p=0.12 |
| *NAMPT* | 0.19 (0.33)  [-0.47-0.86], p=0.55 | 0.39 (0.38)  [-0.38-1.17], p=0.15 | 0.59 (0.39)  [-0.19-1.37], p=0.13 |
| *PTBP1* | 0.04 (0.14)  [-0.25-0.33], p= 0.79 | 0.12 (0.14)  [-0.17-0.42], p=0.42 | 0.16 (0.15)  [-0.16-0.47], p=0.32 |
| *MLST8* | 0.09 (0.12)  [-0.15-0.35], p=0.44 | 0.35 (0.25)  [-0.15-0.86], p=0.16 | 0.44 (0.26)  [-0.07-0.97], p=0.09 |
| *COPS7A* | 0.05 (0.11)  [-0.18-0.27], p=0.68 | 0.22 (0.14)  [-0.07-0.51], p=0.13 | 0.27 (0.14)  [-0.03-0.56], p=0.07 |
